# Scaling Behavior of Ionic Conductance Dependent on Surface Charge Inside a Single-Digit Nanopore

**DOI:** 10.3390/molecules30010191

**Published:** 2025-01-06

**Authors:** Anping Ji, Lang Zhou, Qiming Xiao, Jigang Liu, Wenqian Huang, Yun Yu, Zhengwei Zhang, Junhao Pi, Chenxi Yang, Haoxuan Chen

**Affiliations:** 1 School of Mechanical Engineering, Chongqing Three Gorges University, Chongqing 404100, China; 2 Chongqing Engineering Technology Research Center for Light Alloy and Processing, Chongqing 404100, China

**Keywords:** ionic conductance, potential leakage, ion transport, surface charge

## Abstract

The ionic conductance in a charged nanopore exhibits a power-law behavior in low salinity—as has been verified in many experiments ($G_{0}\propto c_{0}^{\alpha }$)—which is governed by surface charges. The surface charge inside a nanopore determines the zeta potential and ion distributions, which have a significant impact on ion transport, especially in a single-digit nanopore with potential leakage. However, precisely measuring surface charge density in a single-digit nanopore remains a challenge. Here, we propose a methodology for exploring the power-law variation of ionic conductance, with potential leakage taken into account. We conducted experiments to measure the ionic current using silicon nitride nanopores and employed a continuous theory to explore the relationship between pore-bound concentration and surface charges. Considering that the influence of potential leakage on concentration follows a power-law relationship, we established a coefficient (α) to examine the controlling factors of potential leakage and modified the conductance model to obtain the ion mobility inside a nanopore.

## 1. Introduction

The modulation of ionic transport through nanopores can exhibit remarkable physical properties, including high transport efficiency [1], selectivity [2,3], and ionic current rectification [4]. These significant characteristics often play a crucial role and even determine the functionality of potential applications such as biosensors [5], desalination systems [6], supercapacitors and energy converters [7]. Confined electrolyte solutions in nanopores often behave in ways that deviate from the predictions of classical theories. Such deviations are particularly significant in nanopores with pore diameters comparable to the Debye length [8,9]. Furthermore, because of the extremely minute scale of ion transport at the nanoscale, it is challenging to measure its distinctive transport properties directly. Usually, they are determined indirectly by applying the ion transport theory to the measured current data [10,11,12,13,14]. Stein et al. [15] reported that ion transport is governed by surface charge density at low salt concentrations and deviates significantly from bulk behavior; at high salt concentrations, ion transport is consistent with the bulk transport mechanism. Additionally, the ionic conductance demonstrates a power-law variation with increasing concentration [16].To understand the power-law relationship, it is crucial to study the double electric layer formed by the surface charge, which profoundly affects both electrophoresis and electroosmosis in nanopores [17]. Full numerical computation of the Poisson–Nernst–Planck (PNP) equations for ionic motion, which always takes the total charge neutrality inside nanoconfined regions for granted, is widely used in different electrochemical applications, from electroosmotic pumps to energy conversion devices. Charge overspill [18] and electroneutrality breakdown [19,20] have demonstrated the particularity of the electric double layer in charged nanopores, but classical theories do not adequately account for the mechanisms underlying the qualitative features of narrow nanopores. The non-negligible potential leakage or the violation of the electrically neutral assumption in the nanopore are the causes.

**Figure 1:**
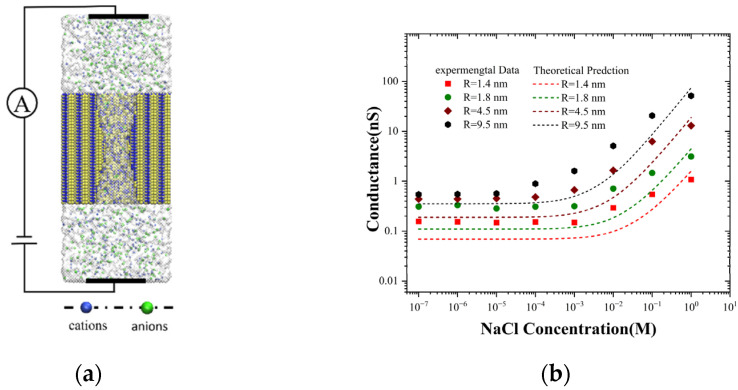
(**a**) The semi-sectional view of ion transport through a nanopore. The silicon nitride film with a nanopore divides the liquid pool into aqueous cis and trans chambers (not to scale). (**b**) The experimental values and theoretical predictions of ionic conductance corresponding to different pore sizes vary in line with concentration (R = 1.4, 1.8, 4.5, 9.5 nm, L = 22, 24, 32, 29 nm).

However, prior theories of conductivity did not take the leakage of potential into account. Here, we propose a methodology for exploring the power-law variation of salt concentration via solid nanopores, with potential leakage taken into account. We measured the ionic current through experiments with silicon nitride nanopores and obtained the ionic conductance following Ohm’s law (Figure 1a), which indicated a certain deviation from the theoretical results. This significant discrepancy is typically attributed to the assumption that the ion concentration is equivalent to the bulk concentration [21]. We employed a continuous theory to explore the relationship between the pore-bound concentration and the surface charge, concluding that the actual concentration within the pore is significantly higher than the bulk concentration, which offers a reasonable explanation for the origin of the discrepancy in the conductance measurement. Considering that the influence of potential leakage on concentration follows a power-law relationship, we established a leakage coefficient to examine the controlling factors of potential leakage. Subsequently, we modified the conductivity model to obtain the ion mobility in nanopores, finding that in nanopores, due to the presence of high electric fields and relatively low permeability, the ion mobility is significantly enhanced, which provides profound insights into the development of novel nano-fluidic devices.

## 2. Results and Discussion

### 2.1. Ion Conductance with Solid-State Nanopores

At low concentrations, ion transport is primarily governed by the distribution and properties of surface charges. These surface charges exert a substantial influence on the movement and trajectory of ions, thereby regulating the entire transport process. Nevertheless, at high concentrations, the performance of ion transport undergoes a remarkable transformation and becomes strikingly similar to that witnessed in the bulk solution (Figure 1b). This phenomenon has been widely acknowledged and established as a consensus in a multitude of meticulously reported studies [22]. Our meticulous experimental results also unambiguously demonstrate this relationship [23,24]. Specifically, concerning ion conductance, it can be observed that as the concentration drops below 1 mM, a gradual approach towards saturation is manifest. Concurrently, when the concentration exceeds 1 mM, a distinct manifestation of ohmic characteristics is detectable. Interestingly, it is worth noting that for nanopores with a radius ranging from 1.4 nm to 4.5 nm, the concentration at which the transition from the surface charge-controlled mode to bulk behavior mode begins is 1 mM. This finding is strikingly consistent with the previously reported results specifically for 2 nm nanochannel [24]. As the pore size continues to increase, in 9.5 nm nanopores, the concentration at which the transition from the surface charge-controlled mode to bulk behavior mode begins drops significantly to 0.1 mM. This observation is completely consistent with the conclusion drawn for microchannels [15]. The variation in the transition concentration from surface charge control mode to bulk behavior mode among different pore sizes underscores the significant influence of pore size on surface charge, warranting careful consideration. This discovery deviates from the theoretical predictions put forward in previous studies, where it was postulated that surface charge remained constant throughout the process. Nevertheless, the current experimental outcomes expose the complexity and variability of this parameter, mandating us to carry out a more comprehensive and meticulous examination.

Many early experimental studies showed that the ionic conductance of a nanopore or channel with a length of *L* and a radius of *R* can be represented as follows [17,21]:

(1) $$G\left(\frac{L}{\pi R^{2}}+\frac{1}{2R}\right)=F{c_{0}(\mu }_{{Na}^{+}}+\mu_{{Cl}^{-}})+\mu_{{Na}^{+}}\frac{{2\sigma }_{s}}{R}$$

where $c_{0}$ and $u_{i}$ are the concentration and the electrophoretic mobility of an ion of the ith specie, respectively; and F is the Faraday constant. For the silicon nitride nanopore, the surface charge can be estimated by the dissociation constant and pH value as $\sigma_{s}=\sigma_{0}K_{d}/(K_{d}+c_{H^{+}})$, where $\sigma_{0}$ is the maximum possible charge density (−1.28 $C\cdot m^{-2}$ in this experiment), and $K_{d}$ is the equilibrium dissociation constant (equal to 10^−6^ M) [16]. At room temperature, when a NaCl solution is exposed to the atmosphere, it assimilates carbon dioxide, consequently giving rise to a solution with a pH value of approximately 5 [23,24]. The mobilities of ${Na}^{+}$ and ${Cl}^{-}$ within solutions of diverse concentrations are obligated to conform to the Kohlrausch law, and the corresponding values can be extracted from the CRC handbook [25]. Hence, we are able to calculate the theoretical predicted conductance for the four disparate geometric parameters in the experiment on the basis of Equation (1), as depicted by the dotted lines in Figure 1b.

**Figure 2:**
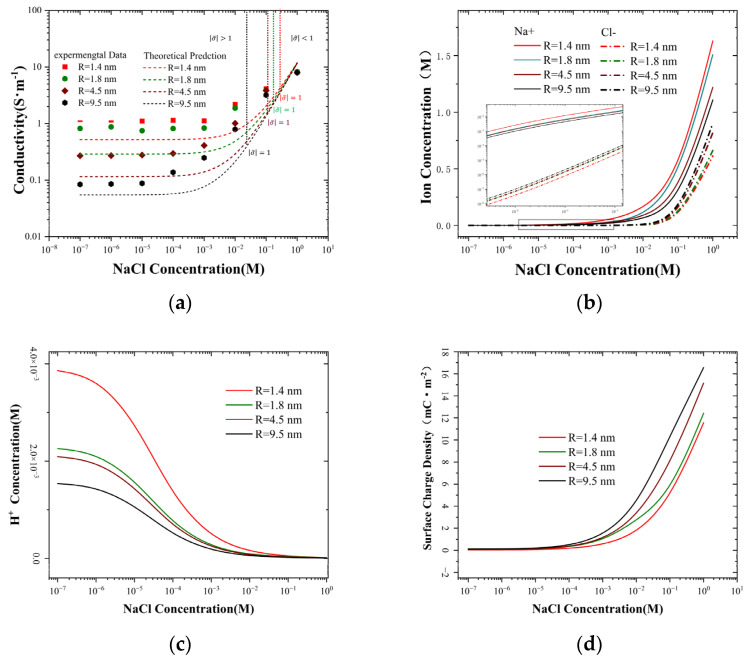
(**a**) The experimental values and theoretical predictions of ionic conductivity corresponding to different pore sizes vary in line with concentration. (**b**) Na^+^/Cl^−^ concentration inside the nanopore. (**c**) H^+^ concentration inside the nanopore. (**d**) Surface charge density inside the nanopore for NaCl/HCl solutions.

The theoretical predictions are in substantial agreement with the experimental results in the ohmic region; however, ion conductance in the surface charge-dominated region and the transition region is lower than the experimental values. The reasons accounting for the deviation between this theory and the experiment are that the average concentration of ionic conductance through nanopores is higher than that of the bulk concentration, and the surface charge varies with the size of the pore. The conductivity data presented in Figure 2a further confirm this deviation. Similar to the conductance, the conductivity in the low salt concentration is arranged by pore size. Nevertheless, the smaller the pore size is, the higher the conductivity becomes. To obtain a deeper understanding of this deviation, further discussion on the relationship between surface charge and ion concentration is requisite.

### 2.2. The Ensemble-Averaged Concentration Inside the Nanopore

Experimental observations have demonstrated that when the surfaces of dielectric nanofluidic channels (such as Al_2_O_3_, Si_x_N_y_) are in contact with aqueous solutions, a charge modulation phenomenon emerges on the wall surfaces. The genesis of this charge modulation is not stochastic but rather a physically regular phenomenon that typically occurs under specific conditions and is influenced by the concentration of salt solutions and the pH value. Especially for silicon nitride nanofluidic channels, the key surface reactions that lead to this charge modulation include the following aspects [21,26]

(2) $$SiOH↔{SiO}^{-}+H^{+}$$

(3) $$SiOH+H^{+}\;↔{SiOH\;}_{2}^{+}$$

Following the recommendations of earlier works and by combining Equations (2) and (3), the surface charge of a nanopore can be calculated as follows [21]:

(4) $$\sigma_{s}=-F\left(\Gamma_{{SiO}^{-}}-\Gamma_{{SiOH}_{2}^{+}}\right)=-F\Gamma_{t}\left\{\frac{10^{-pK_{A}}-10^{-pK_{B}}[H^{+}{]}_{S}^{2}}{10^{-pK_{A}}+[H^{+}{]}_{s}+10^{-pK_{B}}[H^{+}{]}_{+}^{2}}\right\}$$

where $\Gamma_{t}$ is the total number of silanol sites per unit area of the nanopore wall, $[H^{+}{]}_{s}$ is the proton concentration near the surface, $pK_{A}$ and $pK_{B}$ are the equilibrium constants for these reactions *K_A_* and *K_B_*, $pK_{A}=-logK_{A}$ and $pK_{B}$ $=-logK_{B}$, respectively. Considering the electrochemical equilibrium between nanopore and reservoir, for a 1:1 electrolyte solution, the following relationship holds [27]:

(5) $$\mu_{0}+k_{B}T\ln\left(c_{0}\right)=\mu_{0}+k_{B}\mathrm{Tln}\left(c_{P}^{i}\right)\pm e\psi_{p}$$

where $\mu_{0}$ represents the standard chemical potential of ions, $c_{0}$ is the averaged concentration of ion in bulk solution, $\psi_{p}$ is the mean potential in the pore, and $c_{P}^{i}$ is the ensemble-averaged concentration of ion in the nanopore. For a given surface charge density, the ion concentration inside the nanopore must satisfy a certain relationship due to the quasi-electroneutrality condition, as suggested by earlier works:

(6) $${2\pi RL\sigma }_{s}=-\pi R^{2}Le\sum z_{i}c_{P}^{i}$$

where $z_{i}$ represents is the valence of an ion of the ith species. By combining Equations (4)–(6), the surface charge of the ions and the ion concentration within the pores can be obtained, as depicted in Figure 2.

Figure 2b presents the variation of ion concentration within the nanopore in relation to the bulk concentration. When the bulk concentration is lower than 0.1 mM, $c_{P}^{Na^{+}}$ is 10^5^ times greater than $c_{P}^{Cl^{-}}$, and $\;c_{P}^{Cl^{-}}$ is also considerably smaller than $c_{P}^{H^{+}}$, as depicted in Figure 2c. At 1 mM, $c_{P}^{Na^{+}}$ is already higher than $c_{P}^{H^{+}}$, and its concentration is higher than the bulk value. This implies that ion transport is mainly accomplished by Na^+^ and H^+^ at low concentrations, and ion transport is dominated by surface charge. When the bulk concentration exceeds 1 mM, $c_{P}^{Na^{+}}$ begins to take the leading position, and $c_{P}^{Cl^{-}}$ also increases rapidly. For a 1.4 nm nanopore, when the bulk concentration reaches 0.1 M, $c_{P}^{Na^{+}}$ is approximately 20 times greater than $c_{P}^{Cl^{-}}$, and when the bulk concentration is 1 M, $c_{P}^{Na^{+}}$ and $c_{P}^{Cl^{-}}$ are 1.63 M and 0.62 M, respectively. Based on its trend, it can be inferred that when the bulk concentration exceeds 1 M, $c_{P}^{Cl^{-}}$ can be compared to $c_{P}^{Na^{+}}$ and is much higher than $c_{P}^{H^{+}}$,whose contribution to conductivity cannot be ignored. As the bulk concentration rises, Na^+^ and Cl^−^ concentrations concurrently increase, yet the trends diverge. Owing to the reinforcing impact of surface charges on the transport of counterion and the repelling influence on the transport of co-ion, $c_{P}^{Na^{+}}$ manifests an ascending tendency as the radius of nanopores decreases, while $c_{P}^{Cl^{-}}$ rises as the radius of nanopores increases. The trend of $c_{P}^{H^{+}}$ is contrary to Na^+^ and Cl^−^, and it decreases as the bulk concentration rises. At low concentrations, $c_{P}^{H^{+}}$ is significantly higher than $c_{P}^{Na^{+}}$ and $c_{P}^{Cl^{-}}$, and it is also higher than the concentration of H ions in the bulk solution (10^−5^ M). When the bulk concentration ascends to 1 mM, an augmentation in $c_{P}^{Na^{+}}$ induces a decline $c_{P}^{H^{+}}$,thereby reducing the surface protonation and increasing the surface charge density. Analogously to Na^+^, the smaller the nanopore size is, the greater $c_{P}^{H^{+}}$ becomes. The changes in surface charges are determined by the proton concentration within the pores, which can be theoretically predicted from Equation (4) and also reflected in Figure 2d. Interestingly, when the bulk concentration is less than 1 mM, $c_{P}^{H^{+}}$ is higher than the concentration of H+ ions in the bulk solution, resulting in a smaller surface charge value, such as for a nanopore with a radius of 4.5 nm, where the maximum value is 0.9 mC·m^−2^. When the bulk concentration exceeds 1 mM, $c_{P}^{H^{+}}$ drops sharply, the degree of protonation on the surface weakens, and the surface charge strengthens, ultimately reaching 15.2 mC·m^−2^ at 1 M.

### 2.3. Surface Charge with Solid-State Nanopores

**Figure 3:**
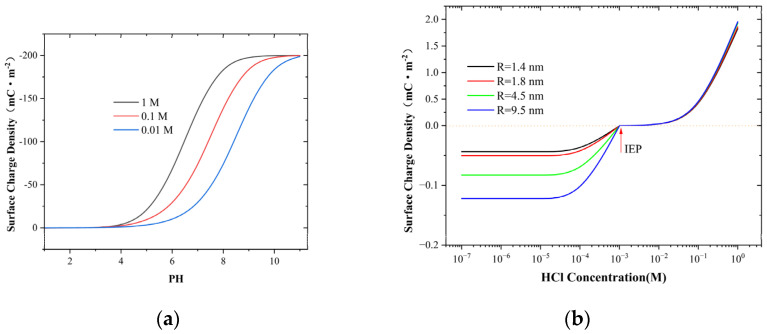
(**a**) Dependence of surface charge density on various pH values in 4.5 nm nanopores. (**b**) The surface charge density of nanopores of different sizes was calculated in relation to the concentration of HCl.

To further explore the relationship between the surface charge and the concentration of the inner surface of the pore, we combined Equations (4) and (5) to compute the variation of the surface charge with the pH value for a 4.5 nm pore, which is presented in Figure 3a. Meanwhile, we also measured the variation of the electrical conductivity of the HCl solution with the concentration (Figure S1b) in the experiment. At low pH values, the concentration of dissolved protons in the bulk solution is high, providing sufficient protons for the protonation reaction of the amphoteric silanol groups on the silicon nitride surface; thus, the surface charge is low. As the pH value decreases from 2 to 1, this protonation reaction causes the surface to change from a negative charge to a small positive charge. For instance, the surface charge value is 0.14 mC·m^−2^ at pH 1 for a 0.1 M NaCl solution, indicating that the surface charge has become positive. This situation is even more prominent in the HCl solution, where the pH value for the change from negative to positive is approximately 3.3 (Figure 3b). This value corresponds to the isoelectric point (IEP) of the silicon nitride surface, which is consistent with previous research findings [25,28].

As shown in Figure 3a, as the pH value increases, the surface charge becomes less than zero and increases in magnitude. The NaCl solution experiences a rapid increase within the pH range of 5 to 8. The surface charge reaches saturation at a pH of 8 in 1 M salt solution, attaining 0.2 mC·m^−2^. Meanwhile, saturation is achieved at a pH of 10 in salt solutions of lower concentrations, indicating that an increase in concentration is conducive to enhancing the occurrence of the deprotonation reaction on the silicon nitride surface, which is similar to the conclusion in Figure S2a. However, in the HCl solution, the surface charge retains saturation after the pH value exceeds 4, which differs from the NaCl solution. Similar to NaCl solution, the larger the pore size in HCl solution, the higher the surface charge. This phenomenon is mainly attributed to the influence of ion concentration within the pore on the surface charge. When $c_{P}^{Na^{+}}$ is small, a higher $c_{P}^{H^{+}}$ is needed to maintain local electrical neutrality, resulting in a lower surface charge value in the NaCl solution. However, in an HCl solution, at low concentrations, the concentration of protons within the pores is significantly higher than that of other ions. This high proton concentration within the pores is detrimental to the deprotonation reaction of amphoteric silanol groups on the surface of silicon nitride. As a consequence, a relatively small negative surface charge is manifested on the surface. When the HCl concentration exceeds 1 mM, the protonation reaction prevails, and the surface charge turns positive.

### 2.4. Electric Potential Leakage with Solid-State Nanopores

Scaling behavior for ionic transport and its fluctuations occur due to the leakage of charge potential into the reservoir, resulting in a deficiency of net charge within the nanopore. Among commonly utilized nanopore materials, leakage is frequently associated with Poole–Frenkel effects. This leakage typically becomes discernible when the electric field exceeds 10^9^ V/m. Nevertheless, due to the finite permittivity of these materials, the electric field lines can penetrate the dielectric film, leading to considerable field leakage [29]. From Equation (5) and the electroneutrality condition, the Donnan potential is described as:

(7) $$\psi_{Donnan}=\frac{k_{B}T}{2e}\log\frac{c_{P}^{-}}{c_{P}^{+}}=\frac{k_{B}T}{e}\log\left(\sqrt{1+{\tilde{\sigma }}^{2}}-\tilde{\sigma }\right)$$

where $\tilde{\sigma }\;$ is a dimensionless coefficient. For a 1:1 electrolyte and homogeneous charge distribution in a cylindrical nanopore, $\tilde{\sigma }\;$ can be written as $\tilde{\sigma }=-\sigma /FRc_{0}$. If there is no leakage of nanopore potential, the Donnan potential should be equal to the average potential inside the pore. After leakage occurs, the potential inside the pore is smaller than the Donnan potential, $\psi_{Donnan}=(1-\alpha )\psi_{P}$, where α is the leakage coefficient. Combining Equations (5) and (7), we can derive the relationship between nanopore concentration and bulk concentration as:

(8) $$c_{P}^{\pm }=c_{0}(\sqrt{1+{\tilde{\sigma }}^{2}}\pm \tilde{\sigma }{)}^{1-\alpha }$$

**Figure 4:**
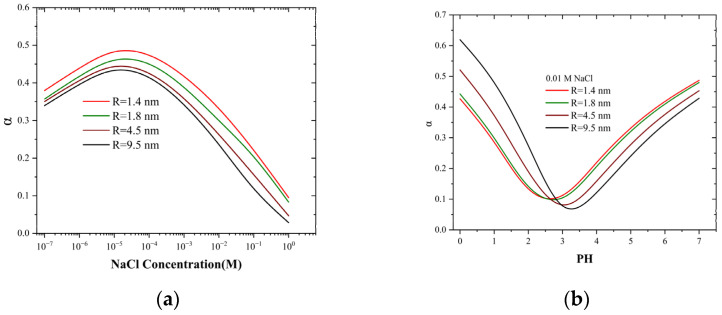
Dependence of the leakage coefficient on (**a**) bulk salt concentration and (**b**) various pH values in 4.5 nm nanopores.

When the coefficient α = 1, $c_{P}^{\pm }$ will be equal to the bulk concentration, indicating that the potential is fully leaking into the liquid pool, which corresponds to the bulk condition. If the coefficient α = 0, there is no potential leakage, and $c_{P}^{\pm }$ is higher than the bulk concentration. At the low concentration limit or small pore sizes, $\left\lceil \tilde{\sigma }\right\rceil \ll 1$, $c_{P}^{\pm }\approx c_{0}$. At the high concentrations limit or large pore sizes, $\left\lceil \tilde{\sigma }\right\rceil \gg 1$, $c_{P}^{+}\approx c_{0}{\left\lceil 2\tilde{\sigma }\right\rceil }^{1-\alpha }\propto {\left\lceil \sigma \right\rceil }^{1-\alpha }$, and $c_{P}^{-}$ approaches zero. The trends of the pore concentrations in these two limiting cases are consistent with the experimental conductivity trends, as shown in Figure 2a. The region $\left\lceil \tilde{\sigma }\right\rceil$ > 1 is dominated by surface charges, while the region $\left\lceil \tilde{\sigma }\right\rceil$ < 1 varies linearly with concentration, similar to the bulk situation. Combining Equation (8), we can further investigate the influence of bulk concentration and PH on the leakage of electrical potential, the results of which are shown in Figure 4.

In nanopores of various sizes, the electrostatic leakage coefficient (α) fluctuates with the change in concentration, attaining an extreme value at approximately 0.01 mM. Intriguingly, this value is comparable to the proton concentration in the bulk solution (Figure 4a). Meanwhile, contrary to the common comprehension, the smaller the pore size, the greater the leakage coefficient. The peak coefficient of the 1.4 nm nanopore is approximately 0.5, indicating that nearly half of the ions are leaking (Figure 4b). The reason is that as the concentration rises, the surface charge also increases; however, the rate of ion depletion is faster. Hence, the surface potential leaks into the liquid pool to capture more ions. This can be observed from the conductivity (Figure 2a) and ion concentration (Figure 2b), where the smaller the pore size, the larger the corresponding values, which also substantiates this conclusion. For a 0.01 M NaCl solution, the pH value has a distinct effect on the electrostatic leakage coefficient compared to the salt concentration. The value initially decreases and then increases; the minimum value is near pH = 3. The combination of Figure 3b reveals that this change occurs because at higher proton concentration (PH < 3), the proton concentration is higher, and the amphoteric silanol groups on the silicon carbide surface undergo extensive protonation reactions, resulting in a positive surface charge. Smaller pore sizes have less surface charge (Figure S2b) and a higher proton concentration, thereby leading to a smaller leakage coefficient. At lower proton concentration (PH > 3), the proton concentration decreases, and the amphoteric silanol groups on the silicon carbide surface undergo extensive deprotonation reactions, resulting in a negative surface charge.

### 2.5. Ionic Conductance Model with Electric Potential Leakage

We further examined the ionic conductance by combining experimental results and the coefficient of leakage, dividing the conductance into two categories: electrophoresis conductance ($G_{Ph}$) and electroosmosis conductance ($G_{eo}$). Moreover, we further subdivided electrophoresis into parts: access conductance ($G_{a}$), nanopore conductance ($G_{p}$), surface conductance ($G_{S}$), and electric-potential leakage conductance ($G_{l}$). which satisfies the relationship, $G_{ph}^{-1}=G_{a}^{-1}+{\left(G_{S}+G_{P}+G_{l}\right)}^{-1}$. Combining earlier research work, the ionic conductance ($G_{0}$) can be written as [12,13,22,30,31]:

(9) $$G_{0}=\sum_{i}F\left\{{c_{i}^{+}(\mu }_{i}^{+}{+\mu_{eo})(\sqrt{1+{\tilde{\sigma }}^{2}}+\tilde{\sigma })}^{1-\alpha }+{c_{i}^{-}(\mu }_{i}^{-}{-\mu_{eo})(\sqrt{1+{\tilde{\sigma }}^{2}}-\tilde{\sigma })}^{1-\alpha }\right\}{\left(\frac{L}{\pi R^{2}}+\frac{1}{2R}\right)}^{-1}$$

**Figure 5:**
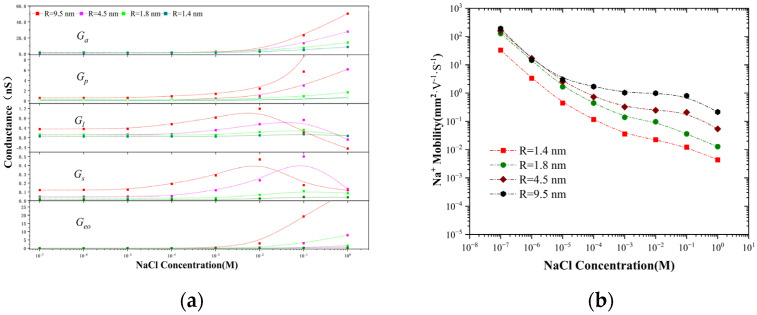
(**a**) Access conductance ($G_{a}$), nanopore conductance ($G_{p}$), surface conductance ($G_{S}$), electric-potential leakage conductance ($G_{l}$), and electroosmotic conductance ($G_{eo}$) of NaCl solution. (**b**) Na^+^ mobility inside the nanopore as a function of reservoir concentration.

Equation (9) expresses the influence of the leakage coefficient on the power-law relationship between conductance and concentration while also providing a clearer expression of the influence of the surface charge on the conductance within the nanopores (Figure S3a,b). When the concentration is above 10 mM, the conductance inside the nanopore is jointly determined by $G_{S}$, $G_{P}$, and $G_{P}$, yet the total sum ($G_{S}+G_{P}+G_{l}$) remains lower than access conductance (Figure 5a). It can be observed that among the subdivided conductance values, the access conductance is the highest, exerting the least hindrance to ion transport. Thus, ions need to queue up to enter the nanopore after reaching the nanopore mouth. This also leads to the concentration polarization phenomenon in the nanopore, which is in accordance with the conclusions of molecular dynamics studies [32]. However, when the concentration is below 1 mM, the ion transport within the nanopore is in the depletion state, and the proton transport within the pore plays a dominant role. The value of the electric potential leakage ($G_{l}$) and the surface potential ($G_{S}$) is greater than the entrance conductance (*G_a_*) and the nanopore conductance ($G_{P}$), which is consistent with the previous theoretical predictions. The electroosmotic conductance only becomes evident at high concentrations, which is consistent with previous research findings [24]. It was also noted that the surface potential ($G_{S}$) and electrical potential leakage ($G_{l}$) reached their maximum values at 0.1 mM because the surface charge value reached its minimum at this concentration, and more Cl ions participated in ion transport, thereby playing a role in increasing the conductance.

The maximum bulk mobility of Na^+^ in NaCl solution under infinite dilution conditions [24] is $5.19\times 10^{-8}\;m^{2}\cdot V^{-1}\cdot s^{-1}$. However, at a bulk concentration less than 10^−5^ M, the Na^+^ mobility inside the nanopore has been higher than this limit value (Figure 5b). At a bulk concentration of 1 M for a 4.5 nm nanopore, the Na^+^ mobility is approximately equal to the maximum bulk mobility under infinite dilution conditions. This increase in mobility requires either a sufficiently high-frequency alternating electric field (Debye–Falkenhagen effect) or an electric field strength above 10^7^ $V\cdot m^{-1}$ (Wien effect) to be achieved. In the nanopore current experiment, the internal potential of the nanopore is indeed higher than this critical electric field strength, thereby causing ions to accelerate in migration. This remarkable phenomenon, through its distinctive manifestation and potential mechanisms, offers new ideas for the design of novel nano-fluidic devices.

## 3. Materials and Methods

In the present investigation, ion transport experiment in single-digit nanopores was devised in line with published research to explore the relationship between ion current and concentration [33,34]. The manufacturing process of single-digit nanopores (R = 1.4, 1.8, 4.5, 9.5 nm) mainly involves chemical deposition, thinning, and perforation of the silicon nitride film (Figure S1). We detected transmembrane ionic currents through the nanopore by inserting two electrodes into a salt solution with a concentration gradient that extended up to the salinity of seawater (10^−7^ M to 1 M). Our investigation centered on the combined effects of surface potential and the nanoconfined environment on electric potential leakage, which we evaluated by measuring the dependence of ionic conductance on concentration. Figure 1a shows that the silicon nitride membrane nanopore divides the liquid chamber into cis. and trans. chambers, filled with a NaCl solution with a concentration ranging from 10^−7^ M to 1 M. A transmembrane voltage is applied to the two ends of the nanoporous membrane, and ionic currents are measured using a patch clamp amplifier. The ionic conductance derived from the recorded I–V curve demonstrates a power-law variation (*G* $\propto c_{0}^{\alpha }$), as indicated by the data points in Figure 1b.

## 4. Conclusions

Ionic conductance is a key parameter for the design of new nanofluidic devices, and it is particularly important to predict it more accurately. Nevertheless, the existing theoretical models for predicting the ionic conductance of single-digit nanopores deviate from the experimental outcomes. This deviation stems from underestimation of the concentration within the pore and disregard of the influence of potential leakage. In this research, we initially measured the ionic current experimentally to obtain the conductance data, which were subsequently compared with the existing theoretical predictions to analyze the disparities. The analysis revealed that ion concentration and surface charge are the key factors causing this difference. Based on the ion protonation and deprotonation at the silicon nitride interface, as well as the requirement of ion transport to satisfy the principle of overall electrical neutrality, we re-examined the changes in ion concentration and surface charge within the pores. We defined a leakage coefficient to characterize the potential leakage in single-digit nanopores and found that the leakage coefficient is controlled by the nanopore size, bulk concentration, and pH. This also gives rise to scaling behavior between the average concentration within the nanopore and the bulk concentration ($c_{P}^{\pm }\propto c_{0}^{\alpha }$), resulting in a proportional conductance ($G_{0}\propto c_{0}^{\alpha }$). By integrating the leakage coefficient and the existing experimental data, we carried out a detailed study of the conductance and found that the access conductance at the high bulk concentration plays a dominant role, which is mainly determined by the bulk concentration. When the bulk concentration is low, the surface charge and surface potential leakage assume the leading role, which is determined by the surface charge. Meanwhile, the ion mobility in the nanopore is much higher than anticipated, especially when the bulk concentration is low. The model presented in this paper offers novel insights for the development and design of new nano-fluidic devices.

## Data Availability

Data are contained within the article and Supplementary Materials.

## Supplementary Materials

The following supporting information can be downloaded at: https://www.mdpi.com/article/10.3390/molecules30010191/s1, Figure S1: (a) Schematic illustration of the experimental devices. The silicon nitride film with nanopore divides the liquid pool into aqueous cis. and trans. chambers. The salt solution contains four species, ${OH}^{-}/{Cl}^{-}/H^{+}/{Na}^{+}$. The ionic transport induces an ionic current through the conical nanopore, the magnitude of which depends on the access, pores, surface charge, electroosmosis and the potential leakage conductance. (b) The experimental values of ionic conductance corresponding to different pore sizes vary in line with concentration; Figure S2: (a) Nanopores were placed in bulk salt solutions with different pH (=5, 7.9, 11), and the surface charge density varies with the bulk concentration. (b) The nanopores of different sizes were placed in 10 mM NaCl solution, and the variation of the surface charge density with the pH of the aqueous solution was observed; Figure S3: (a) The theoretical prediction of the leakage coefficient (0, 0.2, 0.4, 0.6, 0.8, 1) for different conductance under a pore radius of 1.4 nm is presented. (b) Theoretical predictions of the variation of conductivity with concentration under different surface charge conditions. Refs [35,36,37,38,39,40,41,42,43,44] are cited in Supplementary Materials.
