# Supplementary material for: Scaling Behavior of Ionic Conductance Dependent on Surface Charge Inside a Single-Digit Nanopore
Source: Molecules. 2025 Jan 6;30(1):191. doi: 10.3390/molecules30010191 (PMC11721664; doi:10.3390/molecules30010191)
Supplement: Supplementary file 1 [file molecules-30-00191-s001.zip › molecules-3392063-supplementary.pdf]

Supporting Information

Scaling Behavior of Ionic Conductance Dependent on Surface Charge Inside a Single-Digit Nanopore

Anping Ji [1, 2] Lang Zhou [1] Qiming Xiao [1, 2] Jigang Liu [1] Wenqian Huang [1] Yun Yu [1] Zhengwei Zhang [1] Junhao Pi [1] Chenxi Yang [1] Haoxuan Chen [1]

1.School of Mechanical Engineering, Chongqing Three Gorges University, Chongqing, 404100.

2. Chongqing Engineering Technology Research Center for Light Alloy and Processing, Chongqing, 404100

*Correspondence: japmail721@163.com

1. Experiment detail

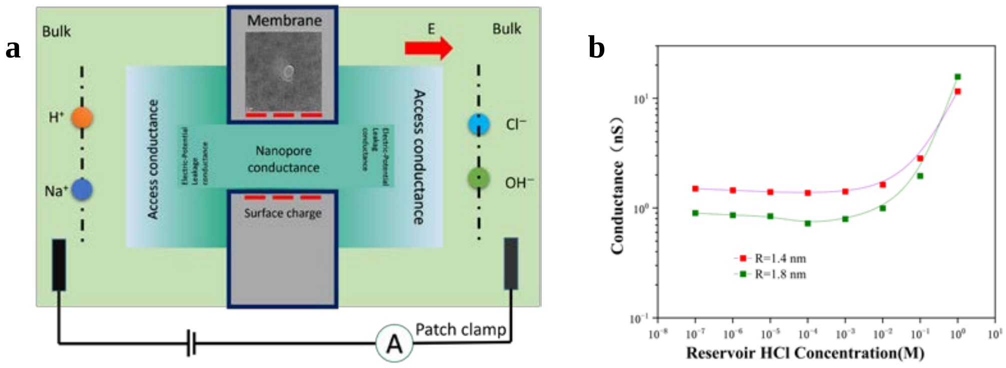

Figure S1. (a) Schematic illustration of the experimental devices. The silicon nitride film with nanopore divides the liquid pool into aqueous *cis.* and *trans.* chambers. The salt solution contains four species, $OH^-/Cl^-/H^+/Na^+$.The ionic transport induces an ionic current through the conical nanopore, the magnitude of which depends on the access, pores, surface charge, electroosmosis and the potential leakage conductance. (b) The experimental values of ionic conductance corresponding to different pore sizes vary in line with concentration.

To investigate the alterations in surface charge density within nanopores, we fabricated a series of silicon nitride thin films of diverse sizes by adhering to the methodologies established in previous research[1-3]. A Si_3N_4 membrane was cultivated on a silicon wafer through low-pressure chemical vapor deposition. Subsequently, an opening was created on the opposite side of the wafer via a wet etching process to expose the silicon nitride thin film. After the etching process, the silicon nitride film was exposed to a high-energy focused ion beam to decrease its thickness. The final stage entailed drilling a nanopore using a transmission electron microscope (TEM) operating at 300 kV. The size of the fabricated nanopore could be modulated using a low-intensity electron beam. Before conducting the measurement experiment, the chips were subjected to a rigorous cleaning procedure in piranha solution at 120 °C for 1 hour to remove surface contaminants and enhance pore hydrophilicity. Subsequently, the chips were rinsed three times with deionized water to ensure thorough cleanliness.The Si_3N_4 membrane containing the nanopores divides the liquid pool into aqueous cis and trans compartments. Ag/AgCl electrodes are immersed on both sides of the pore and connected to a patch clamp amplifier (HEKA EPC 10 USB,

HEKA Instruments) to measure the ionic current with picoampere sensitivity. Subsequently, a degassed and filtered salt solution is added to the liquid pool and allowed to reach equilibrium before commencing the experiment. By sweeping the voltage from -500 mV to 500 mV at a scan rate of 100 mV every 2 seconds, numerous sets of current data can be collected. During the measurement, the bulk concentration is gradually increased from low to high, ranging from 10^{-7} M to 1 M.

2. The ensemble averaged concentration inside the nanopore

Since the dielectric material of the membrane wall in contact with an aqueous solution exhibits a charge-regulated nature, its surface charge density can be determined by the following deprotonation/protonation reactions with equilibrium constants K_A and K_B.

$$SiOH \leftrightarrow SiO^- + H^+ \tag{S1}$$

$$SiOH + H^+ \leftrightarrow SiOH_2^+ \tag{S2}$$

In the aforementioned reactions, $10^{-pK_A} = \Gamma_{SiO^-}[H^+]_s/\Gamma_{SiOH}$ and $10^{-pK_B} = \Gamma_{SiOH_2^+}/([H^+]_s\Gamma_{SiOH})$. Γ_{SiOH}, Γ_{SiO^-}, and $\Gamma_{SiOH_2^+}$ represent the surface site densities of $SiOH$, SiO^-, and $SiOH_2^+$ respectively, and $[H^+]_s$ is the proton concentration near the surface. The total number site density of dissociable MOH molecules on the membrane wall surface is $\Gamma_{TOTAL} = \Gamma_{SiOH} + \Gamma_{SiO^-} + \Gamma_{SiOH_2^+}$. The surface charge density of the membrane wall can be expressed as follows:

$$\sigma_s = -F\left(\Gamma_{SiO^-} - \Gamma_{SiOH_2^+}\right) = -F\Gamma_t\left\{\frac{10^{-pK_A} - 10^{-pK_B}[H^+]_S^2}{10^{-pK_A} + [H^+]_s + 10^{-pK_B}[H^+]_+^2}\right\} \tag{S3}$$

For the silica-based nanopore/nanochannel, the values reported for the parameters Γ_{TOTAL}, pK_A and pK_B fall respectively within the ranges of 3.8-8 nm^{-2}, 6.6-8, and 0-2[4].

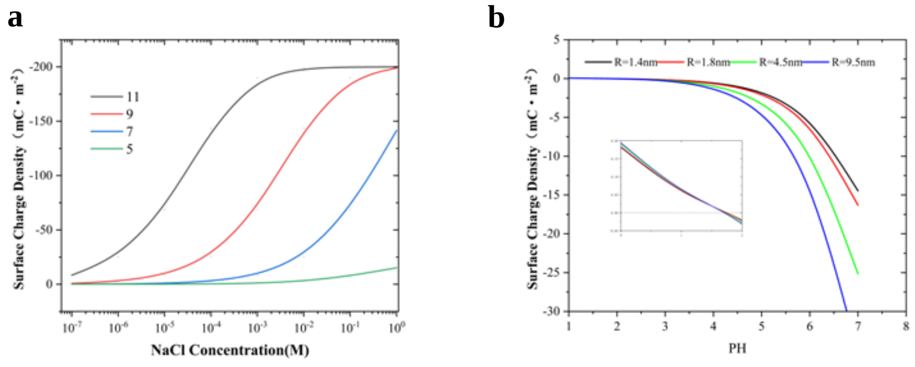

Figure S2. (a) Nanopores were placed in bulk salt solutions with different pH (=5,7.9,11), and the surface charge density varies with the bulk concentration. (b) The nanopores of different sizes were placed in 10 mM NaCl solution, and the variation of the surface charge density with the pH of the aqueous solution was observed.

To further understand the role of concentration in ion transport behavior, we combined equation 5 to estimate the ion concentration inside the nanochannel. Here, we define the ensemble average concentration as the average concentration of all ions in the access conductance[5], surface conductance, potential leakage conductance[6], and nanopore conductance regions. $n_{OH^-}/n_{Cl^-}/n_{H^+}/n_{Na^+}$ are used to represent the concentration of $OH^-/Cl^-/H^+/Na^+$, which are different from bulk concentration n_0.For a given surface charge density σ, the ion concentration inside the nanopore must satisfy a certain relationship due to the quasi-electroneutrality condition, as suggested by earlier works[7]: $\frac{\sigma}{eR} = n_{OH^-} + n_{Cl^-} - n_{H^+} - n_{Na^+}$ .As we performed the measurements at ambient conditions, the pH of

electrolyte solution was 5 due to CO_2 absorption[7, 8]. By using the Donnan equilibrium condition to relate electric potentials and Poisson-Boltzmann theory, we can get the result of ion concentration, as shown in Figure S2.

4. Theoretical model for conductance

many experiments study ion dynamics in micron-sized channel, and conductance measurement (for neutral pores) is estimated as[9, 10] $G_P = \sum_i Fc_i \mu_i \left(\frac{L}{\pi R^2}\right)^{-1}$. However, the conductance (G_p) only is a reasonably accuracy for channels ($L \gg R$) with a very large aspect ratio L/R, where the access resistance is negligible. When the ratio of film thickness to pore size is small ($L \sim R$), the influence of entrance resistance on system resistance cannot be ignored[11-15]. Theoretically, the access conductance(G_a) was derived by Hall[16] and can be expressed as $G_a = \sum_i Fc_i u_i 4R$ .Ionic conductance of ions can be theoretically predicted through a combination of access resistance and pore resistance as suggested by earlier works[17],$G_0 = \sum_i Fc_i \mu_i \left(\frac{L}{\pi R^2} + \frac{1}{2R}\right)^{-1}$.

For the charged nanopore, the surface conductance make an additional contribution to the entire conductance, as nanopores requires counterions to screen the surface charge and maintain electrical neutrality[18, 19]. The surface conductance(G_s) can be expressed as $G_a = \mu_s \frac{2\pi\sigma R}{L}$, where μ_s is the mobility of counterions of a charged pore surface[5]. The entire conductance(G_0) can be written as $G_0^{-1} = G_a^{-1} + (G_S + G_P)^{-1}$. By combining equations 1 and 2, the total conductance can be expressed as[6]

$$G_0 = \sum_i Fc_i \mu_i \sqrt{1+\tilde{\sigma}^2} \left(\frac{L}{\pi R^2} + \frac{1}{2R}\right)^{-1} . \tag{S4}$$

A large number of experiments have shown the failure of the principle of electrical neutrality inside the nanopore region, such as charge overspill, end effects, the surface-electric-potential Leakage and electroneutrality breakdown, but the entire nanopore system remains electroneutrality [6, 20-23]. The reason for these phenomena is that the surface charge potential leaks into the reservoir and the net charge inside the pores is insufficient. This potential leakage is different from the effect of surface charges, which enhances the counterion transport and weakens the co-ion transport. Taking the electric potential leakage conductance (G_l) into consideration, the electrophoretic conductance (G_{ph}) can be expressed as

$$G_{ph} = \sum_i F\left\{c_i^+ \mu_i^+ (\sqrt{1+\tilde{\sigma}^2} + \tilde{\sigma})^{1-\alpha} + c_i^- \mu_i^- (\sqrt{1+\tilde{\sigma}^2} - \tilde{\sigma})^{1-\alpha}\right\} \left(\frac{L}{\pi R^2} + \frac{1}{2R}\right)^{-1} \tag{S5}$$

where α is the fraction of the surface-electric potential that leaks out of the pore[6]. The total conductance of the nanopore should be composed of two parts: electrophoresis conductance (G_{ph}) and electroosmotic conductance (G_{eo}) affected water transport driven by the ionic migration. Then, the entire conductance (G_0) can be written as[6, 18]

$$G_0 = \sum_i F\left\{c_i^+ (\mu_i^+ + \mu_{eo}) (\sqrt{1+\tilde{\sigma}^2} + \tilde{\sigma})^{1-\alpha} + c_i^- (\mu_i^- - \mu_{eo}) (\sqrt{1+\tilde{\sigma}^2} - \tilde{\sigma})^{1-\alpha}\right\} \left(\frac{L}{\pi R^2} + \frac{1}{2R}\right)^{-1} \tag{S6}$$

4 ion mobility

Electroosmotic mobility. The electroosmotic mobility derived by Biesheuvel and Bazantusing SC theory and the Donnan equation can be expressed as[6, 24]

$$\mu_{eo} = \frac{-\sigma R}{4\eta} \tag{S7}$$

where η is the viscosity of the solution. η can be obtained from the Stokes equation.

Ion mobility. In this paper, we assume that the transport of different ions in the nanopore has a similar relationship with the transport of bulk behavior. For fully dissolved sodium chloride solution, ionic mobility in a bulk solution can be obtained, as suggested by earlier works:

$$\mu_{bulk} = \frac{\mu_{bulk}}{1+\frac{0.508*\sqrt{I_Z}}{3.29*\alpha\sqrt{I_Z}}} \tag{S8}$$

The above equation applies to ambient temperature at 25℃, $\mathbf{I_z} = \frac{1}{2}\sum_j c_j^2$ is the ionic strength, and α is an adjustable parameter related to the ion size (Cl-, a ≈ 0.3 nm; Na+, a ≈ 0.4 nm)[7]. Combining Equations S6-S9, we can obtain the variation of ionic conductivity with concentration, as shown in Figure S3. In the investigation of the effect of surface potential leakage on ionic conductivity, the parameters of the nanopore were set to R=1.4nm and L=15nm; the bulk concentration of NaCl solution, was varied from 10-7 to 100M; σ was set to 5mC/m^2, and α, the leakage coefficient, was set to 0, 0.2, 0.4, 0.6, 0.8, and 1, respectively, and the results were calculated. Figure S3a shows the changes in the nanopore value in two regions: one is when $|\tilde{\sigma}|$ is less than 1, in which the conductance is linearly related to the concentration and consistent with the bulk behavior of the solution; the other is when $|\tilde{\sigma}|$ is greater than 1, in which the conductance tends to saturate. It is worth noting that as α increases from 0 to 1, the conductance in the region dominated by surface charges exhibits a trend that the larger the surface potential leakage coefficient, the closer the conductance is to the bulk behavior. When α = 1, the conductance and concentration are linearly related and also indicate that the surface potential is fully leaked into the liquid reservoir. When α = 0, surface charges dominate in the low concentration region, in which case the surface potential is fully acting within the nanopore and does not leak into the liquid reservoir.

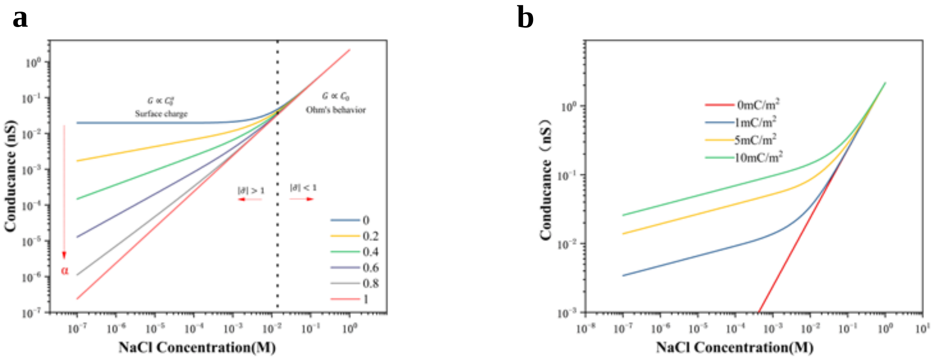

Figure S3. (a) The theoretical prediction of the leakage coefficient (0,0.2,0.4,0.6,0.8,1) for different conductance under a pore radius of 1.4nm is presented. (b) Theoretical predictions of the variation of conductivity with concentration under different surface charge conditions.

References

[1] Zhan L, Jin T, Zhou J, *et al.* Fast Probing Amyloid Polymorphism Via Nanopore Translocation[J]. Nano Letters, 2023, **23**(21): 9912-9919.

[2] Lin K, Chen C, Wang C, *et al.* Fabrication of Solid-State Nanopores[J]. NANOTECHNOLOGY, 2022, **33**(27): 272003.

[3] Ma J, Li K, Li Z, *et al.* Drastically Reduced Ion Mobility in a Nanopore Due to Enhanced Pairing and Collisions between Dehydrated Ions[J]. JOURNAL OF THE AMERICAN

CHEMICAL SOCIETY, 2019, **141**(10): 4264-4272.

[4] Yeh L H, Zhang M K,Qian S Z. Ion Transport in a Ph-Regulated Nanopore[J]. Analytical Chemistry, 2013, **85**(15): 7527-7534.

[5] Luan, Binquan. Numerically Testing Phenomenological Models for Conductance of a Solid-State Nanopore[J]. Nanotechnology, 2015, **26**(5): 055502.

[6] Noh Y, Aluru N R R. Ion Transport in Electrically Imperfect Nanopores[J]. ACS Nano, 2020, **14**(8): 10518-10526

[7] Duan C, Majumdar A. Anomalous Ion Transport in 2-Nm Hydrophilic Nanochannels[J]. Nature Nanotechnology, 2010, **5**(12): 848.

[8] Van d H, Frank H. J., Stein D,Dekker C. Streaming Currents in a Single Nanofluidic Channel[J]. Physical Review Letters, 2005, **95**(11): 116104.

[9] Tsutsui M, He Y, Furuhashi M*, et al.* Transverse Electric Field Dragging of DNA in a Nanochannel[J]. Scientific Reports,

[10] Smeets R M M, Keyser U F, Krapf D*, et al.* Salt Dependence of Ion Transport and DNA Translocation through Solid-State Nanopores[J]. Nano Letters, 2006, **6**(1): 89-95.

[11] Garaj S, Hubbard W, Reina A*, et al.* Graphene as a Subnanometre Trans-Electrode Membrane[J]. Nature, 2010, **467**(7312): 190-193.

[12] Hyun, Rollings, Ryan*, et al.* Probing Access Resistance of Solid-State Nanopores with a Scanning-Probe Microscope Tip[J]. Small, 2012, **8**(3): 384-384.

[13] Sahu S, Zwolak M. Maxwell-Hall Access Resistance in Graphene Nanopores[J]. Physical Chemistry Chemical Physics, 2018, **20**(7): 4646-4651.

[14] Sahu S, Zwolak M P. Access Resistance in Atomically Thin Nanopores[J]. Biophysical Journal, 2018, **114**(3): 493a.

[15] Antonio, Alcaraz, M.*, et al.* Ion Transport in Confined Geometries Below the Nanoscale: Access Resistance Dominates Protein Channel Conductance in Diluted Solutions[J]. Acs Nano, 2017 Oct 24, **11**(10): 10392-10400.

[16] Hall, J. E. Access Resistance of a Small Circular Pore[J]. Journal of General Physiology, 1975, **66**(4): 531-532.

[17] Lee C, Joly L, a A S*, et al.* Large Apparent Electric Size of Solid-State Nanopores Due to Spatially Extended Surface Conduction[J]. Nano Letters, 2012, **12**(8): 4037-4044.

[18] Chen Y F, Ni Z H, Wang G M*, et al.* Electroosmotic Flow in Nanotubes with High Surface Charge Densities[J]. Nano Letters, 2008, **8**(1): 42-48.

[19] Chaudhury S, Bhattacharyya A,Goswami A. Electrodriven Ion Transport through Crown Ether-Nafion Composite Membrane: Enhanced Selectivity of Cs+ over Na+ by Ion Gating at the Surface[J]. Industrial & Engineering Chemistry Research, 2014, **53**(21): 8804-8809.

[20] Luo Z X, Xing Y Z, Ling Y C*, et al.* Electroneutrality Breakdown and Specific Ion Effects in Nanoconfined Aqueous Electrolytes Observed by Nmr[J]. Nature Communications, 2015, **6** 6358.

[21] Gupta A, Shim S, Issah L*, et al.* Diffusion of Multiple Electrolytes Cannot Be Treated Independently: Model Predictions with Experimental Validation[J]. Soft Matter, 2019, **15**(48): 9965-9973.

[22] Hashemi Amrei S M H, Bukosky S C, Rader S P*, et al.* Oscillating Electric Fields in Liquids Create a Long-Range Steady Field[J]. Physical Review Letters, 2018, **121**(18): 185504.185501-185504.185506.

[23] Jiang D E, Jin Z,Wu J. Oscillation of Capacitance inside Nanopores[J]. Nano Letters, 2011, **11**(12): 5373-5377.

[24] Biesheuvel P M, Bazant M Z. Analysis of Ionic Conductance of Carbon Nanotubes[J]. PHYSICAL REVIEW E, 2016, **94**(5): 050601.
